# NIH Investment in HIV and Implementation Science

**DOI:** 10.1002/jia2.70148

**Published:** 2026-10-08

**Authors:** Geri R. Donenberg, Daniel E. Murphy, Amber Wilson, Robert C. Cregg, Melissa Herrera, Mohini Bhakta, Hayatt Mohamed, Mary T. Glenshaw

**Affiliations:** ^1^ Office of AIDS Research National Institutes of Health Rockville Maryland USA

**Keywords:** HIV, implementation science, NIH, portfolio analysis, research support, strategic planning

## Abstract

**Introduction:**

Research supported by the National Institutes of Health (NIH) has revolutionized HIV prevention and treatment, but not all people have benefited from these advancements. Implementation science seeks to bridge the gap between a discovery and when it is applied in the real world, by elucidating the methods that promote the adoption, uptake and integration of evidence‐based strategies into routine care. Disparities in who benefits from the advancements have prompted calls to accelerate HIV implementation science. This paper provides a snapshot of research funded by the NIH at the intersection of HIV and implementation science.

**Methods:**

The NIH Office of AIDS Research conducted portfolio analyses using the Information for Management Planning Analysis and Coordination (IMPAC II) database; the Research, Condition, and Disease Categorization (RCDC) system; and manual coding to describe select award characteristics. We examined awards coded as both RCDC categories HIV and Dissemination and Implementation Research (D&IR) from fiscal years (FY) 2021–2024, followed by more detailed analyses of awards made in FY2024.

**Results:**

NIH invested USD 697 million in 1569 awards at the intersection of HIV and D&IR from FY2021–2024. During this period, funding for D&IR and HIV increased by 65%. In FY24 alone, 407 awards were made in D&IR and HIV, representing 7% of the total NIH HIV budget appropriated by Congress. Led by the National Institute of Mental Health, support for D&IR in HIV was represented by 15 NIH institutes, centres and offices. Among a subset of 226 FY24 awards consisting of R‐mechanisms, studies spanned the HIV prevention and treatment cascade and focused on multiple age groups. Forty‐one percent of the awards included hybrid effectiveness‐implementation designs, and the Consolidated Framework for Implementation Research (CFIR) was the most frequently cited framework (*n* = 82; 37%). Over 93% of awards employed both quantitative and qualitative methods.

**Conclusions:**

The NIH investment in HIV‐related implementation science is growing, but it made up a small portion (7%) of the total HIV budget in FY24. Challenges characterizing awards on key implementation science factors underscore opportunities to strengthen the science. These data support NIH's commitment to accelerate implementation research to end HIV and ensure its full public health benefit.

## 1 Introduction

Over the past 40 years, since the first AIDS diagnosis, the vast majority of NIH‐funded research sought to characterize the virus, identify transmission pathways, understand disease progression, develop diagnostic tools, and search for antiretroviral therapies [1, 2, 3, 4, 5]. These investments yielded tremendous impacts and biomedical discoveries that prevent HIV transmission, halt or slow viral progression, offer a functional cure, and treat the disease. A person diagnosed with HIV today faces similar challenges as people with other chronic diseases, such that if managed carefully, they can live a near‐normal lifespan. In 2026, we have the tools to prevent HIV transmission, stop disease progression, and suppress viral load, accomplishments that would not have been possible without NIH‐funded research [6, 7].

Yet, we are at a crossroads. Despite these remarkable scientific advances, HIV incidence has remained stubbornly stable. In the United States, 43% of Americans with HIV are not virally suppressed, increasing the likelihood of disease progression and onward transmission. The most recent data from the CDC indicate that 13% of Americans with HIV are unaware of their status, and for those who know their status, 34% are not receiving care [8]. The prevalence of HIV drug resistance in the United States varies by drug class and population, with a reported 19% frequency of at least one transmitted drug resistance mutation in recent years [9, 10, 11]. Furthermore, over 52% of incident diagnoses were in the Southern United States, and 20%–25% occurred among women, people under age 35 years and from heterosexual activity [8]. Taken together, these data underscore the continued challenge of bringing an end to HIV in the United States.

All United States citizens deserve to benefit from NIH‐funded scientific discoveries, but evidence shows that we are not reaching communities with the most HIV prevention, treatment, and care needs [12, 13, 14]. These disparities are explained in large part by reduced access to services, persistent stigma, health‐system challenges, low provider awareness, and diverse social and structural determinants of health that impede intervention uptake and adoption [14, 15, 16, 17]. These obstacles must be overcome to achieve an end to the HIV epidemic.

Changes in the nature of the HIV epidemic must be met with changes in science to address the most pressing concerns. The protracted amount of time between a discovery and its application in practice has spurred extensive research to understand the causes of and solutions to closing the gap [18]. Implementation science, in particular, can elucidate methods and strategies needed to reduce implementation barriers by identifying and addressing drivers of uptake and adoption of evidence‐based HIV interventions and promoting attitudes, capacity, and behaviour that improve implementation outcomes [19, 20]. Implementation science can also elucidate how to strengthen fidelity to effective interventions and sustain and scale up evidence‐based strategies to reach populations with the greatest need [21, 22, 23]. Ultimately, the pace at which research discoveries are moved into real‐world settings will depend on a variety of factors (e.g. intervention characteristics, clinical specialty, resources) that implementation research is poised to and should examine [24].

Recognizing the evolving nature of the HIV epidemic, the NIH HIV Research Program is accelerating investments in implementation science to optimize and extend HIV research's impact. Initial NIH investments in the *Ending the HIV Epidemic in America* initiative since 2019 led to important successes in increased HIV testing, linkage to care, pre‐exposure prophylaxis (PrEP) prescriptions and viral suppression, particularly in the Southern United States [16, 25, 26, 27]. While the NIH‐supported HIV research portfolio remains robust and comprehensive (i.e. two‐thirds of the congressionally mandated budget for HIV is dedicated to a broad scientific agenda), NIH is poised to expand its support for implementation research to ensure all people benefit from the scientific advances achieved with NIH funding.

This paper describes three analyses of awarded grants that represent the intersection of dissemination and implementation research (D&IR) and HIV research from fiscal years (FY) 2021–2024. We present trends across awards and funding, and describe select characteristics of a subset of projects, including the percentage of awards focused on different aspects of the HIV cascade, population HIV status and age groups, study designs, and implementation science frameworks.

## 2 Methods

### 2.1 Search Strategy

The NIH Office of AIDS Research (OAR) conducted portfolio analyses of funded awards from FY 2021–2024 using two sources: the Information for Management, Planning, Analysis, and Coordination (IMPAC II) database [28] and the Research, Condition, and Disease Categorization (RCDC) system [29]. The study period comprises October 2020 (FY21) through September 2024 (FY24). Ethical review and a waiver of informed consent were not required as the data used in the present analyses do not involve human subjects. Apart from NIH award application data used for manual coding, all other data are publicly available. Award metadata included application ID, activity code, whether the application responded to a specific solicitation (e.g. Notice of Funding Opportunity or NOFO), the investigator's status as early stage, the NIH Institute, Center, and/or Office (ICO) that administered the award, the ICO that funded the award, and reportable categorical funding. The portfolio was defined as all awards and reportable funding categorized by the NIH RCDC system as both categories: HIV and D&IR.

### 2.2 Screening and Eligibility

Awards were screened for three separate analyses. First, we identified all HIV and D&IR awards from FY21−24 to examine trends over time. Next, we created a subset of awards categorized as both HIV and D&IR from FY24 only, the most recent year of available data. Finally, we sought to characterize select features of a subset of the FY24 HIV and D&IR awards using manual coding where variables were not otherwise captured. For this subset of FY24 awards, we restricted the dataset to five activity codes: R00, R01, R03, R21 and R34, representing investigator‐led projects. We excluded 244 of the 470 HIV and D&IR awards that were research career programmes (K‐series), cooperative agreements (U‐series), research programme projects and centres (P‐series), institutional training and director programme projects (D‐series), research infrastructure and conference grants (R13, R24, R33, R61), fellowship programmes (F‐series), research and development‐related contracts (N‐series), resource programmes (G‐series) and training programmes (T‐series) (see Figure 1).

**FIGURE 1:**
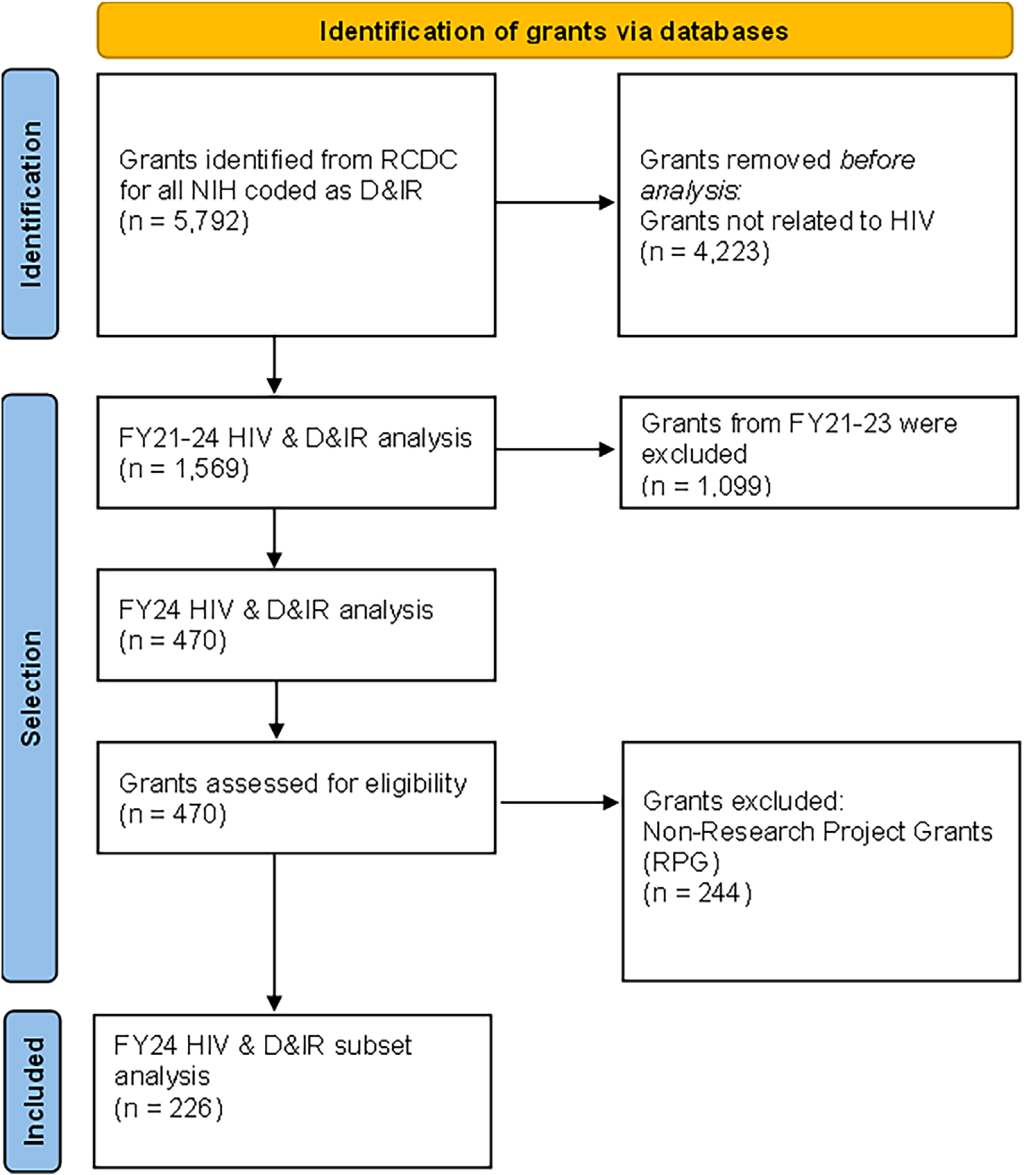
PRISMA flow diagram of grant identification, selection, and inclusion from the NIH HIV and Dissemination & Implementation Research (D&IR) portfolio, Fiscal Years (FY) 2021–2024.

### 2.3 Abstraction and Coding

For the subset of 226 awards coded manually, analysts from the NIH OAR developed a codebook to characterize seven categories made up of 42 variables. Five analysts read each award abstract, specific aims, and human subjects’ eligibility inclusion/exclusion criteria to code according to the 42 variables defined in the codebook. Each variable was coded as a binary yes or no, or not specified. For some categories, binary variables are also reported if the project included more than one group (e.g. people living with and without HIV). Category and variable definitions are presented in Table 1.

**TABLE 1:** Codebook definitions of categories and variables of manually coded awards from the NIH HIV and Dissemination & Implementation Research portfolio, Fiscal Year 2024 (*n* = 226).

Category	Variable	Definition
HIV status	People living with HIV	Focused on people living with HIV, including activities, services, interventions, or research centred on people with an HIV diagnosis (e.g. antiretroviral therapy [ART], retention/adherence support, care navigation).
	People living without HIV	Focused on people who do not have HIV, including activities, services, interventions or research for this population (e.g. pre‐exposure prophylaxis [PrEP], HIV testing, prevention education).
HIV prevention and treatment cascade	HIV testing	Assessments of HIV testing, screening, or identification.
	Linkage/initiation	Research on initiating HIV prevention (e.g. PrEP, post‐exposure prophylaxis [PEP]), treatment (e.g. ART) or care (e.g. linkage to PrEP/PEP, from HIV testing to HIV prevention or treatment).
	Care	Promotion of engagement in HIV care (e.g. routine clinical visits, case management, lab monitoring).
	Treatment	Evaluations of HIV treatment, ART initiation, viral suppression and/or treatment optimization.
	Retention/adherence	Aimed at keeping people in HIV care or supporting adherence to HIV prevention or treatment, (e.g. retention programmes, ART/PrEP adherence counselling, HIV medication reminders).
Comorbid condition	Projects that indicate assessment of comorbidities, coinfections, or complications that co‐occur in people with or impacted by HIV (e.g. cardiovascular disease, hypertension, viral hepatitis) or complicate HIV acquisition risk, prevention, or treatment (e.g. smoking, drug use, poverty, food insecurity).	
Population age	Under 18 years‐old	Study population is under 18‐years‐old.
	18‐years & older	Study population is equal to or older than 18‐years‐old.
	50‐years & older^b^	Study population has a specific focus on people equal to or older than 50‐years‐old.
Implementation science framework^a^	ADAPT‐ITT [30] Conceptual Framework for Sustainability of Public Health Programs [31] Conceptual Model of Evidence‐based Practice Implementation in Public Service [32] Consolidated Framework for Implementation Research (CFIR) [33] Diffusion of Innovations [34] Diffusion of Innovations in Service Organizations [35] Dynamic Sustainability Framework [36] Exploration, Preparation, Implementation, Sustainment (EPIS) Framework [37] Framework of Public Health Program Capacity for Sustainability (PHPCS) [38] Interactive Systems Framework (ISF) [39] Organizational Theory of Innovation Implementation [40] Practical, Robust Implementation and Sustainability Model (PRISM) [41] PRECEDE‐PROCEED [42] Proctor's Implementation Outcomes Framework [43] Promoting Action on Research Implementation in Health Services (PARIHS) [44] Reach, Effectiveness, Adoption, Implementation, Maintenance (RE‐AIM) [45] Replicating Effective Programs (REP) [46] Shediac‐Rizkallah and Bone's Program Sustainability Framework [47] Stirman Adaptation Framework [48] Theoretical Domains Framework (TDF) [49] Theory of Organizational Readiness for Change [50]	
Study design	Hybrid I	Primary focus on clinical effectiveness while exploring implementation.
	Hybrid II	Equal focus on clinical effectiveness and implementation.
	Hybrid III	Primary focus on implementation with minimal focus on clinical outcomes.
Research methods	Qualitative	Words, observations and experiences to understand meaning, context and perspectives.
	Quantitative	Numerical data that can be counted and analysed.

^a^ Table S1 lists Implementation Science Frameworks in the Fiscal Year 2024 subset by frequency.

^b^ Variable “50‐years & older” may be double coded with variable “18‐years & older” and are not mutually exclusive.

### 2.4 Interrater Reliability

Several steps were used to establish interrater reliability for the manual coding. First, each analyst independently rated the same five awards and then met as a team to discuss and adjudicate discrepancies, reach consensus, update the data, and add additional detail to the codebook. This process was completed three times, with five unique awards each time, for a total of 15 projects, until the analysts achieved a strong consensus. To evaluate final agreement across coders, each analyst was randomly assigned 50 or 51 projects to code independently. Of these, 16 were randomly assigned to a second analyst to formally assess interrater reliability on 19% of the sample (*n* = 40). Overall, the percent agreement at the level of the seven categories ranged from 78% to 100%.

### 2.5 Data Analyses

Datasets for the three levels of analyses were stored in Microsoft Excel. Descriptive summary statistics were calculated for each level in Microsoft Excel (version 2502) and Tableau Desktop (version 2024.2.6) software [51, 52]. HIV funding is a specific determination indicating that congressionally appropriated funds were used to support the award [53]. The number and percentage of awards and the amount and percentage of reportable HIV funding were calculated based on award metadata (i.e. administering ICO, funding ICO, activity code, whether the application responded to a specific solicitation or was investigator‐initiated, the status of the investigator as early stage) and coded categories and variables. Unique application IDs for FY24 HIV and D&IR awards were entered into iSearch, NIH's portfolio analysis platform developed by the Office of Portfolio Analysis to provide curated and linked datasets on NIH awards [54].

## 3 Results

### 3.1 FY21−24 HIV and D&IR Awards

From FY21−24, NIH funded 1569 awards categorized as both HIV and D&IR by the RCDC system. Data show steady growth in both funding awarded and the number of studies over time at the cross‐section of HIV and D&IR (see Figure 2). Total funding increased each year, rising from about $131 million in FY21 to over $216 million in FY24, an increase of 65%. Over the same period, the number of awards climbed consistently, from 307 awards in FY21 to 470 awards in FY24, an increase of 53%. Relative to the full HIV budget appropriation from FY21−24 totalling $12.9 billion, the investment in HIV and D&IR was only $697 million, or 5.4% of the total HIV budget.

**FIGURE 2:**
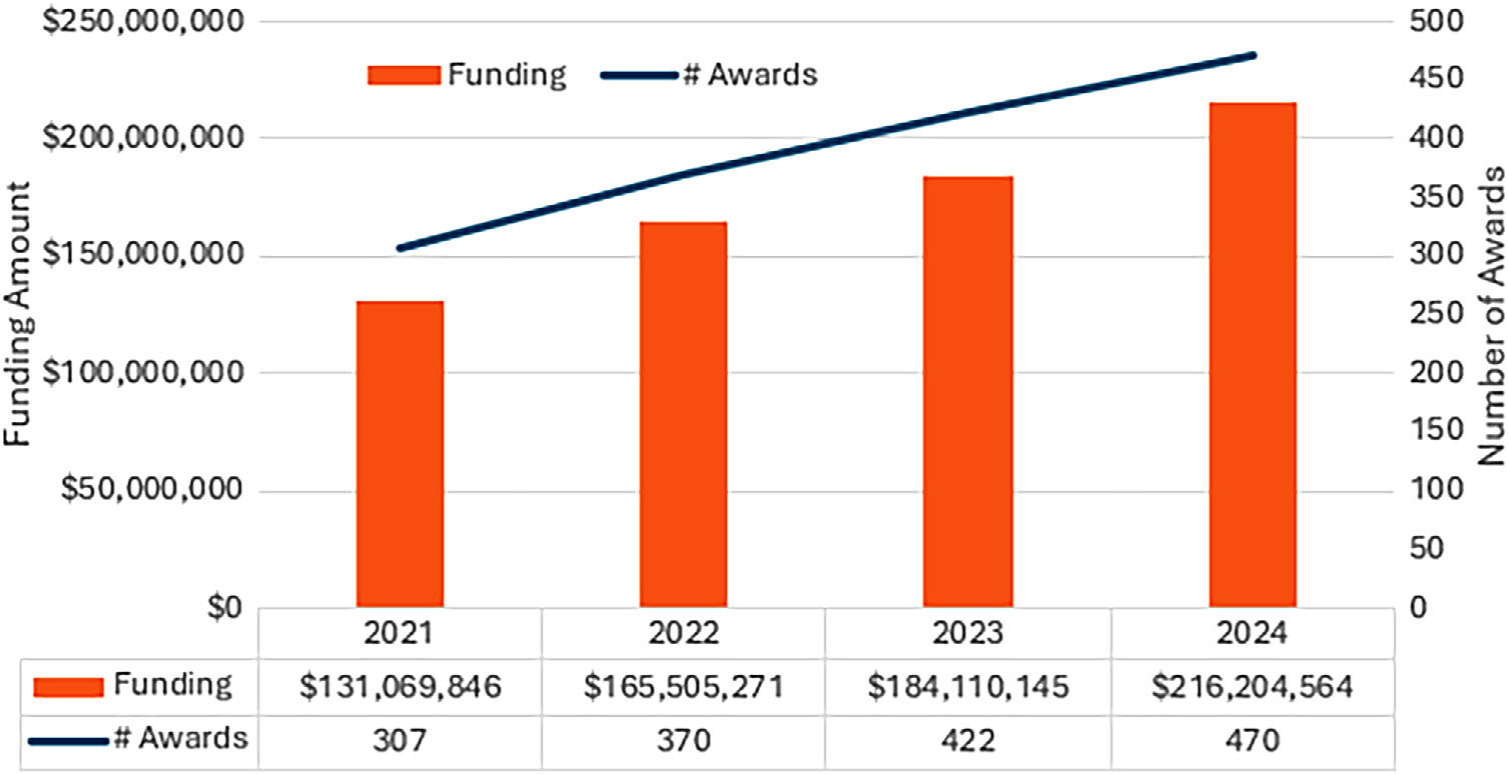
NIH HIV and Dissemination & Implementation Research portfolio by number of awards and annual funding level, Fiscal Years 2021−2024.

Regarding the specific grant mechanisms of the 1569 awarded studies, the distribution shows that R01s accounted for the most significant proportion (32%) of the portfolio from FY21−24 (see Figure 3). The R34 and P30 mechanisms each represented 9%, followed by lower proportions among other mechanisms such as K23, K01, D43 and U01.

**FIGURE 3:**
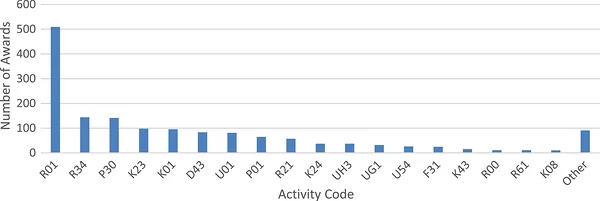
Distribution of grant mechanisms in the NIH HIV and Dissemination & Implementation Research portfolio, Fiscal Years (FY) 2021−2024. Table S2 lists activity codes funded in FY 2021–2024, HIV/AIDS and Dissemination and Implementation Research portfolio.

Finally, there was broad support for HIV and D&IR research across the NIH Institutes, Centres and Offices (ICOs) from FY21−24 (Table 2). Fifteen ICOs contributed funding to these awards. The National Institute of Mental Health both administered the largest proportion of awards (*n* = 555) and invested the most HIV funding in D&IR ($195 million).

**TABLE 2:** Funding for NIH HIV and Dissemination & Implementation Research portfolio by Institute, Center and Office, Fiscal Years 2021−2024, and Fiscal Year 2024 only.

	FY 21−24				FY 24			
Funding ICO	FY 21−24 Funding for HIV and D&IR	Percent of total funding for HIV and D&IR	FY 21−24 HIV funding	Percent of ICO HIV funding for HIV and D&IR	FY 24 funding for HIV and D&IR	Percent of total funding for HIV and D&IR	FY 24 HIV funding	Percent of ICO HIV funding for HIV and D&IR
NIMH	$195,092,896	28%	$778,561,000	25%	$61,246,189	28%	$199,584,000	31%
NIDA	$118,772,774	17%	$1,088,479,000	11%	$32,749,963	15%	$278,533,000	12%
NIAID	$117,601,739	17%	$7,459,536,000	2%	$37,514,102	17%	$1,911,364,000	2%
NCI	$57,628,771	8%	$1,002,920,998	6%	$21,200,367	10%	$256,734,000	8%
NICHD	$55,902,665	8%	$600,751,000	9%	$20,849,559	10%	$152,881,000	14%
NHLBI	$44,535,039	6%	$359,901,000	12%	$9,749,781	5%	$92,953,000	10%
NIAAA	$31,700,252	5%	$136,966,428	23%	$9,409,300	4%	$35,718,831	26%
NINR	$23,285,879	3%	$67,947,999	34%	$7,635,867	4%	$17,375,000	44%
FIC	$21,865,825	3%	$101,286,000	22%	$6,620,914	3%	$25,919,000	26%
NIMHD	$13,534,132	2%	$97,718,000	14%	$4,640,721	2%	$24,982,000	19%
OD	$10,779,992	2%	$576,785,808	2%	$3,179,977	1%	$145,370,000	2%
NIDCR	$2,545,427	<1%	$78,893,995	3%	$883,899	<1%	$20,174,000	4%
NIA	$1,458,610	<1%	$106,464,000	1%	$280,527	<1%	$28,538,000	1%
NIBIB	$1,399,471	<1%	$7,634,000	18%	$0	0%	$1,954,000	0%
NIDDK	$786,354	<1%	$149,057,000	1%	$243,398	<1%	$38,699,000	1%
NINDS	$0	0%	$161,145,834	0%	$0	0%	$41,206,000	0%
NLM	$0	0%	$30,740,000	0%	$0	0%	$7,685,000	0%
NIEHS	$0	0%	$21,870,213	0%	$0	0%	$5,512,000	0%
NIAMS	$0	0%	$19,050,000	0%	$0	0%	$4,875,000	0%
NIDCD	$0	0%	$8,844,999	0%	$0	0%	$2,262,000	0%
NCCIH	$0	0%	$2,840,000	0%	$0	0%	$796,000	0%
**Total**	**$696,889,826**	**100%**	**$12,863,007,113**	**5%**	**$216,204,564**	**100%**	**$3,293,114,831**	**7%**

*Note*: The bold values indicate the totals per column.

Abbreviations: D&IR, dissemination & implementation research; FY, fiscal year; ICO, Institute, Center, Office.

### 3.2 FY24 HIV and D&IR Awards

The analysis of the FY24 HIV & D&IR awards included 470 projects representing a total of $216 million, or 6.5% of the HIV budget appropriation. The awards were funded by 14 ICOs with funding ranging from 28% to <1% of the ICOs HIV allocation. The National Institute of Mental Health dedicated 31% of their HIV budget to D&IR and accounted for 28% of the total funds spent on HIV and D&IR across the NIH (Table 2). The National Institute of Allergy and Infectious Diseases spent 2% of their HIV funding on D&IR, but given the size of their budget, this amounted to 17% of the funds spent on HIV and D&IR in FY24.

Lastly, we used the iSearch Grants module to identify additional NIH spending categories assigned to each of the 470 awards. The spending categories reflect scientific domains and conditions (e.g. comorbidities, research topics) classified by RCDC and represented in iSearch; the list is not exhaustive of all RCDC categories. Figure 4 shows the co‐occurring spending categories for FY24 along with HIV and D&IR; the size of the section is proportional to the number of awards in each category. Notably, the most prevalent categories were health disparities (50%), mental health (41%), women's health (40%), substance misuse (36%), comparative effectiveness research (30%), primary healthcare (15%) and sexually transmitted infections (12%). Awards are commonly assigned to multiple RCDC categories, because many research projects address co‐occurring conditions with HIV. Category percentages are not mutually exclusive and sum to greater than 100%.

**FIGURE 4:**
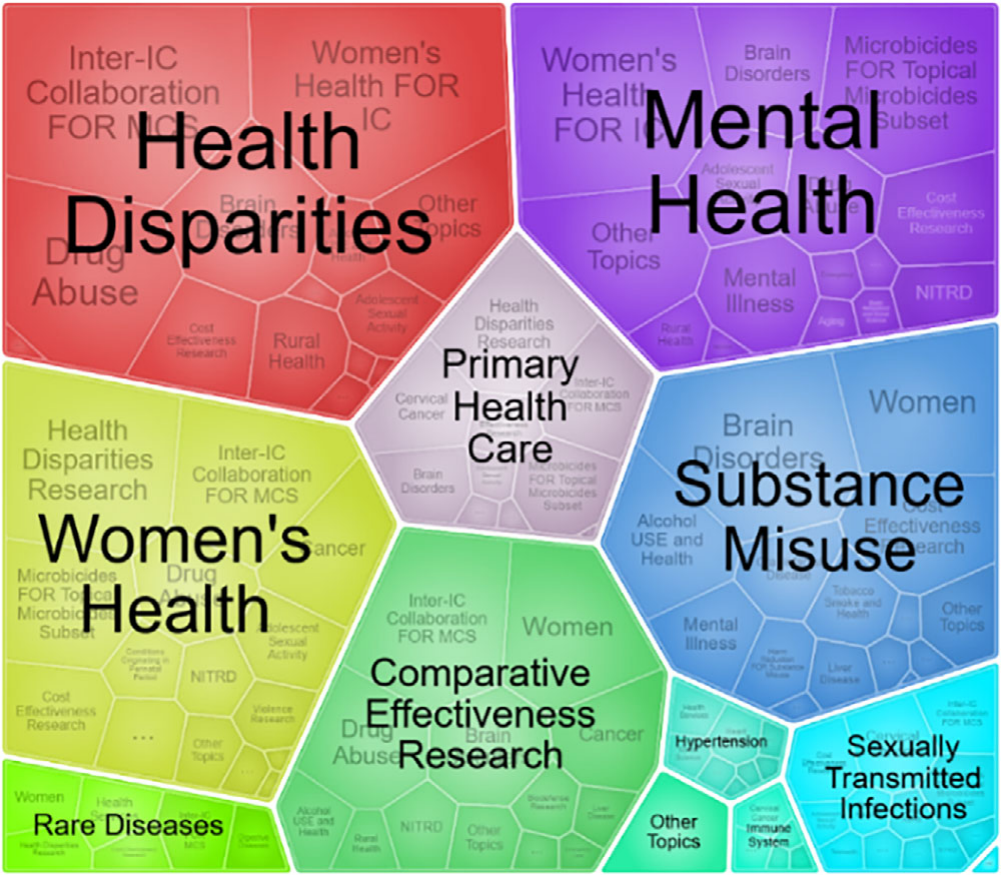
Co‐occurring spending categories in the NIH HIV and Dissemination & Implementation Research portfolio, Fiscal Year 2024.

### 3.3 FY24 HIV and D&IR Awards Subset (*n* = 226)

Of the 470 awards granted in FY24, 226 (48%) represented the five specified activity codes (R00, R01, R03, R21, R34), accounting for an investment of $119 million. These projects included 170 (75%) R01 awards, 35 (15%) R34 awards, 16 (7%) R21 awards, 3 (1%) R00 awards and 2 (1%) R03 awards. Over 80% of the awards in this subset (*n* = 186) were matched to a specific funding opportunity by the ICOs, including Program Announcements (PA), Program Announcements with Special Receipt, Referral, and/or Review Considerations (PAR), Requests for Applications (RFA) and Program Announcements with Set‐Aside Funds (PAS) issued in FY 2018−2024. Of the awards in this same subset of 226 awards, 29% were in response to funding opportunities with a specific set‐aside dollars (*n* = 66). This same subset of 226 awards was matched to 73 funding opportunities, 44% (*n* = 32) of which provided set‐aside funding. These notices of funding opportunity (NOFOs) with set‐aside funding were sponsored by eight ICOs. Sixteen of the 32 NOFOs with set‐aside funding had shared interest from multiple ICOs. Notably, 17 awards (8%) were granted to early stage investigators, although this underestimates the actual number of awards to new investigators, because the career development series (K‐series, D‐series) was excluded from the analysis. The subset of 226 projects were awarded by 10 ICOs, with the National Institute of Mental Health awarding the most HIV and D&IR projects (110, 49%) (see Table 3).

**TABLE 3:** Proportional distribution of NIH HIV and Dissemination & Implementation Research portfolio, Fiscal Year 2024 by total, subset, and ICO.

Admin ICO	Total HIV D&IR awards number; percent	Subset HIV D&IR awards number; percent	Proportion of HIV D&IR awards in ICO HIV portfolio
NIMH	159; 34%	110; 49%	69%
NIDA	66; 14%	40; 18%	61%
NIAID	55; 12%	19; 8%	35%
NICHD	35; 7%	11; 5%	31%
FIC	41; 9%	10; 4%	24%
NIAAA	27; 6%	10; 4%	37%
NINR	11; 2%	10; 4%	91%
NCI	49; 10%	9; 4%	18%
NHLBI	17; 4%	4; 2%	24%
NIMHD	8; 2%	3; 1%	38%
NIA	1; <1%	0; 0%	0%
NIDCR	1; <1%	0; 0%	0%
Total	470; 100%	226; 100%	48%

Abbreviations: D&IR, dissemination & implementation research; ICO, Institute, Center, Office.

### 3.4 Characteristics of the Subset of Awards

Finally, manual coding revealed several features of the subset of awards.

#### 3.4.1 HIV Status and Age

Among the 226 awards, 40% (*n* = 90) focused exclusively on people living with HIV, and 33% (*n* = 74) focused exclusively on people living without HIV. Thirty‐three awards (15%) included both populations. Surprisingly, 29 (13%) projects did not specify the HIV status of participants. Most of the projects (*n* = 213; 94%) focused on adults aged 18‐years‐old and older. Only 54 (24%) included people under 18‐years‐old, and nine (4%) focused on participants aged 50 years and older. Six (3%) projects did not specify population age, and 53 projects included people from more than one age group.

#### 3.4.2 HIV Prevention and Care Cascade

About 65% of funded studies (*n* = 146) focused on retaining people in HIV care and supporting adherence to HIV prevention or treatment. Almost half of the awards (*n* = 108; 48%) examined linkage to and/or initiation of HIV prevention, treatment and care services. While 33% examined HIV treatment, ART initiation, or viral suppression (*n* = 75), 33% focused on receipt of ongoing HIV care (*n* = 75), and 25% focused on HIV testing (*n* = 56). Notably, 27 (12%) did not specify the study's point along the HIV cascade. Most of the awards (*n* = 158, 70%) focused on multiple parts of the cascade. Five awards studied five components of the HIV care cascade, 13 awards studied four, 62 awards studied three, 78 awards studied two and 41 awards studied one.

#### 3.4.3 Implementation Science Framework

There were 36 unique frameworks identified across the 226 awards. The two most cited frameworks were the Consolidated Framework for Implementation Research (CFIR) (*n* = 83, 37%) and the Reach, Evaluation, Acceptability, Implementation, Maintenance (RE‐AIM) framework (*n* = 77, 34%). Other widely cited frameworks included Proctor's Implementation Outcomes Framework (*n* = 33, 15%); Exploration, Preparation, Implementation, Sustainment (EPIS) (*n* = 23, 10%); ADAPT‐ITT (*n* = 23, 10%), and Practical, Robust Implementation and Sustainability Model (PRISM) (*n* = 11, 5%). Importantly, 30 other frameworks were cited in applications, and no specific framework was indicated in 27 projects. Eighty‐seven awards (38%) named more than one framework, and 71 studies reported two frameworks.

#### 3.4.4 Study Design

Most awards did not indicate a hybrid design (*n* = 133; 59%). Where one was reported, 55 (24%) indicated a hybrid type I design, 28 (12%) reported a hybrid type II design, and 10 (4%) stated a hybrid type III design.

#### 3.4.5 Methodology

Almost all the awards planned to employ both quantitative and qualitative research methods (*n* = 211; 93%). Only 10 (4%) planned to use qualitative methods exclusively, and five (2%) reported quantitative methods exclusively.

## 4 Discussion

This paper offers a snapshot of NIH‐funded research at the intersection of HIV and D&IR over 4 years (FY21−FY24), culminating in a closer examination of FY24. Findings reveal several observations concerning the growth, breadth, and depth of the HIV and implementation science portfolio and the differences across ICOs in their funding commitments. While the NIH investment in D&IR and HIV has grown since FY21 by both the number of awards and the amount allocated, the relative proportion of HIV funding used for D&IR is low—just 7% in FY24. Moreover, the investment in D&IR is considerably smaller relative to HIV funding for all other areas of inquiry, including basic discovery, preclinical science and clinical research. These findings support the need to accelerate the investment in D&IR and HIV science to ensure all people benefit from NIH's research investments.

Interest in funding D&IR projects to end HIV is increasing with recognition that we have the tools to prevent and treat HIV. Indeed, these findings show broad support across the ICOs for D&IR research in HIV. With a stated commitment from the NIH Director to accelerate research in implementation science [55], NIH is poised to create new opportunities to push this agenda forward. That said, recent changes in the grant application process and grant‐making procedures pose challenges to increasing the investment quickly and must be addressed. For example, one strategy is to employ grant mechanisms that are nimble and flexible to fast‐track investment opportunities. Challenge Prizes [56] and contracts are two such approaches that could be leveraged to accelerate the pace of funding.

Findings revealed a variety of mechanisms used to pursue HIV and D&IR research. Importantly, the majority were R01 awards, indicating larger‐scale multi‐year investments per project. Recent NIH priorities seek to emphasize and promote investigator‐led science rather than science driven by NIH programme staff [55]. To this end, investigators can consider Highlighted Topics issued by NIH ICOs that describe high‐priority research areas, including HIV implementation science, to encourage investigator‐initiated applications as well as applying for awards through targeted funding opportunities. In the subset analyses, a somewhat limited number of R‐series awards were granted to early stage investigators, underscoring the need to amplify efforts to strengthen the workforce and build capacity to conduct research in HIV and D&IR, key priorities for NIH.

Characteristics of the projects yielded interesting patterns. That 40% of awards used hybrid effectiveness‐implementation designs indicates that the research community remains invested in determining both intervention effectiveness and implementation outcomes in concert [57, 58]. Furthermore, the prominence of women's health and health disparity research as co‐occurring domains with HIV underscores the role of implementation science in bridging known gaps in populations disproportionately affected by HIV. Finally, the spending categories document NIH's commitment to address the full HIV cascade and the variety of comorbid conditions that increase vulnerability to HIV acquisition and exacerbate poor clinical outcomes for people living with HIV, including mental health, substance misuse and sexually transmitted infections.

It is important to note that manual coding posed several challenges reflecting difficulties with the state of the science. The application of implementation science to HIV research suffers from inconsistencies in terminology, definitions and categorization [59, 60]. Core constructs such as implementation outcomes (e.g. feasibility, adoption, sustainment) are often used inconsistently with intervention approaches (e.g. task shifting, community delivery) and implementation strategies (e.g. training, resources and support) [43, 61, 62]. The introduction of new intervention modalities, including long‐acting PrEP and ART, HIV self‐testing, and digital adherence and retention tools, further complicates categorization by blending clinical, behavioural and systems‐level components. These challenges impede comparability across studies and efforts to reach consensus during manual coding. In several publications, implementation scientists highlight the need for increased harmonization across projects and standardized measures that can be used broadly across populations to strengthen the knowledge base and cross‐study comparisons [21, 23, 63, 64, 65]. The NIH is poised to address these challenges in future research. Indeed, the FY2026–2030 NIH Strategic Plan for HIV and HIV‐Related Research urgently calls for greater investment in implementation science to end the epidemic through improved understanding of strategies to implement evidence‐based interventions.

Limitations of the current analysis warrant consideration. RCDC categorical spending data have known challenges that limit certain interpretations of analyses [66]. As RCDC does not prorate the budget of an award across categories, the funding levels of grants analysed may overestimate the implementation science component of HIV awards. In this case, the actual amounts dedicated to implementation science may be less. Further, the system is subject to various classification errors and potential miscalculation of the universe of HIV and D&IR grants. Many awards are multi‐focal, and the relative proportion of implementation research aims were not addressed in the present analyses. Similarly, manual coding can be imprecise, although in achieving consensus, we sought to maximize validity. Finally, we restricted manual coding to a specific subset of R‐series awards. The inclusion of other mechanisms might yield other patterns.

## 5 Conclusions

The NIH investment in HIV and D&IR has grown over the past 4 years, but it still makes up a small proportion of funded awards in the HIV research portfolio. To realize the full public health benefit of scientific advances in HIV, future studies must address how best to reach populations in greatest need, improve adoption of innovations by health systems and scale effective strategies broadly. Future research in HIV and D&IR can inform the science itself by strengthening rigour, reproducibility and transparency—core tenets of scientific excellence and values of the NIH [67].

## Author Contributions

GRD conceptualized the idea and wrote the manuscript; MTG conducted initial analyses and contributed to writing and interpretation of findings; RCC contributed to analysis, methods and results; DEM conducted analysis, contributed to manual coding, wrote portions of the methods and results; MB contributed to analysis and manual coding; HM contributed to manual coding; MH contributed to manual coding; AW contributed to manual coding, writing and interpretation of findings.

## Conflicts of Interest

All authors have no conflicts of interest to declare.

## Supporting information




**Table S1**: Full list of Implementation Science Frameworks coded in analytic subset, FY 2024.
**Table S2**: Activity codes funded in FY 2021–2024, HIV/AIDS and Dissemination and Implementation Research portfolio.

## Data Availability

These data were derived from the following resources available in the public domain: Portfolio Online Reporting Tools (RePORT) https://report.nih.gov/) and RePORT Expenditures and Results (RePORTER) (https://reporter.nih.gov/). Other data used in the manual coding, including the grant‐specific aims and project narratives, are not publicly available.
