# Supplementary material for: NIH Investment in HIV and Implementation Science
Source: J Int AIDS Soc. 2026 Oct 8;29(Suppl 5):e70148. doi: 10.1002/jia2.70148 (PMC13650396; doi:10.1002/jia2.70148)
Supplement: Supplementary file 1 [file JIA2-29-e70148-s001.docx]

**Supplementary Materials:**

**NIH Investment in HIV/AIDS and Implementation Science**

Geri R. Donenberg^1§^, Daniel E. Murphy^1^, Amber Wilson^1^, Robert C. Cregg^1^, Melissa Herrera^1^, Mohini Bhakta^1^, Hayatt Mohamed^1^, Mary T. Glenshaw^1^

1	Office of AIDS Research, National Institutes of Health, Rockville, MD

**Supplementary Table 1: Full list of Implementation Science Frameworks coded in analytic subset, FY 2024**

| Implementation Science Frameworks coded (n=226 awards) | Number of Projects |
| --- | --- |
| CFIR | 83 |
| RE-AIM | 77 |
| Proctor's Implementation Outcomes Framework | 33 |
| EPIS | 23 |
| ADAPT ITT | 23 |
| PRISM | 11 |
| Framework for Reporting Adaptations and Modifications-Enhanced (FRAME) | 6 |
| Theoretical Framework of Acceptability | 5 |
| Expert Recommendations for Implementing Change (ERIC) | 4 |
| Health Equity Implementation Framework (HEIF) | 4 |
| Implementation Research Logic Model (IRLM) | 4 |
| PARIHS | 3 |
| Interactive Systems Framework | 3 |
| Framework for Reporting Adaptations and Modifications to Evidence-Based Implementation Strategies (FRAME-IS) | 3 |
| Theoretical Domains Framework (TDF) | 2 |
| Diffusion of Innovations | 2 |
| IM Adapt Model | 2 |
| Stirman's Adaptation Framework | 1 |
| Program Sustainability Assessment Tool (PSAT) | 1 |
| PRECEDE/PROCEED | 1 |
| Conceptual Model of Evidence-based Practice Implementation in Public Service Sectors (Aarons) | 1 |
| "A scoping study of frameworks for adapting public health evidence-based interventions" publication by Escoffery. | 1 |
| Barker’s Theory for Scale-Up | 1 |
| Cultural adaptation framework | 1 |
| Ecological framework for effective implementation | 1 |
| Framework for Going to Full Scale | 1 |
| Framework of Implementability of Healthcare Interventions | 1 |
| Model for Adaptation Design and Impact (MADI) | 1 |
| Nielsen’s Framework for Usability | 1 |
| PRACTical planning for Implementation and Scale-up (PRACTIS) | 1 |
| Proctor Framework for Intervention Specification | 1 |
| Properties, Actions, Rules, Time, and Environment (PARTE) framework | 1 |
| Systems Analysis and Improvement Approach (SAIA) | 1 |
| The Process Evaluation Framework for Complex Interventions (Bonell's process evaluation framework) | 1 |
| Weiner’s Organizational Theory for Implementation Effectiveness | 1 |
| WHO theory of scale-up | 1 |

**Supplementary Table 2: Activity codes funded in FY 2021–2024, HIV/AIDS and Dissemination and Implementation Research portfolio.**

| Activity Code | Number of Awards | Funding |
| --- | --- | --- |
| R01 | 509 | $319,238,427 |
| R34 | 144 | $34,872,579 |
| P30 | 141 | $43,408,542 |
| K23 | 98 | $17,911,887 |
| K01 | 96 | $15,142,398 |
| D43 | 83 | $21,682,823 |
| U01 | 81 | $92,998,084 |
| P01 | 65 | $16,935,964 |
| R21 | 57 | $12,716,482 |
| K24 | 37 | $6,175,249 |
| UH3 | 37 | $22,889,706 |
| UG1 | 32 | $24,878,607 |
| U54 | 26 | $2,861,635 |
| F31 | 25 | $1,146,874 |
| K43 | 15 | $1,075,145 |
| R00 | 11 | $2,465,747 |
| R61 | 11 | $9,294,582 |
| K08 | 10 | $1,714,240 |
| R33 | 8 | $2,219,258 |
| U24 | 8 | $11,815,642 |
| UG3 | 8 | $4,974,314 |
| R13 | 7 | $2,755,000 |
| UM2 | 7 | $5,201,943 |
| G11 | 5 | $509,783 |
| R25 | 5 | $1,060,687 |
| R37 | 5 | $3,767,774 |
| DP2 | 4 | $3,372,498 |
| K99 | 4 | $507,649 |
| P50 | 4 | $439,995 |
| R03 | 4 | $376,368 |
| T32 | 4 | $1,454,673 |
| F32 | 3 | $228,102 |
| K76 | 2 | $480,661 |
| N01 | 2 | $3,508,343 |
| R24 | 2 | $2,175,701 |
| R44 | 2 | $1,028,068 |
| Y01 | 2 | $825,000 |
| DP1 | 1 | $544,250 |
| F30 | 1 | $53,536 |
| R56 | 1 | $41,105 |
| U19 | 1 | $1,147,426 |
| ZIA | 1 | $993,079 |
